# Remarkable similarities of chromosomal rearrangements between primary human breast cancers and matched distant metastases as revealed by whole-genome sequencing

**DOI:** 10.18632/oncotarget.5951

**Published:** 2015-10-02

**Authors:** Man-Hung Eric Tang, Malin Dahlgren, Christian Brueffer, Tamara Tjitrowirjo, Christof Winter, Yilun Chen, Eleonor Olsson, Kun Wang, Therese Törngren, Martin Sjöström, Dorthe Grabau, Pär-Ola Bendahl, Lisa Rydén, Emma Niméus, Lao H. Saal, Åke Borg, Sofia K. Gruvberger-Saal

**Affiliations:** ^1^ Division of Oncology and Pathology, Department of Clinical Sciences, Lund University, Lund, Sweden; ^2^ Division of Surgery, Department of Clinical Sciences, Lund University and Skåne University Hospital, Lund, Sweden; ^3^ Department of Pathology, Skåne University Hospital, Lund, Sweden; ^4^ CREATE Health Strategic Center for Translational Cancer Research, Lund University, Lund, Sweden

**Keywords:** breast cancer, metastasis, whole-genome sequencing, chromosomal rearrangements, chromothripsis

## Abstract

To better understand and characterize chromosomal structural variation during breast cancer progression, we enumerated chromosomal rearrangements for 11 patients by performing low-coverage whole-genome sequencing of 11 primary breast tumors and their 13 matched distant metastases. The tumor genomes harbored a median of 85 (range 18-404) rearrangements per tumor, with a median of 82 (26-310) in primaries compared to 87 (18-404) in distant metastases. Concordance between paired tumors from the same patient was high with a median of 89% of rearrangements shared (range 61-100%), whereas little overlap was found when comparing all possible pairings of tumors from different patients (median 3%). The tumors exhibited diverse genomic patterns of rearrangements: some carried events distributed throughout the genome while others had events mostly within densely clustered chromothripsis-like foci at a few chromosomal locations. Irrespectively, the patterns were highly conserved between the primary tumor and metastases from the same patient. Rearrangements occurred more frequently in genic areas than expected by chance and among the genes affected there was significant enrichment for cancer-associated genes including disruption of TP53, RB1, PTEN, and ESR1, likely contributing to tumor development. Our findings are most consistent with chromosomal rearrangements being early events in breast cancer progression that remain stable during the development from primary tumor to distant metastasis.

## INTRODUCTION

Disseminated metastases remains the primary cause of mortality for cancer patients, and in breast cancer, this is typically due to metastatic spread to the brain, bones, lung, and/or liver. Presently, metastatic breast cancer is essentially incurable and can arise many years or even decades after treatment of the primary tumor. It is presumed that macroscopic metastatic tumors arise from very slow growing micrometastases that remain subclinical during the asymptomatic years, or from dormant cancer deposits that are triggered into active proliferation by unknown signals or events. It is not well understood how similar the breast cancer metastases are to the primary tumors from which they originate, to what degree tumor evolution continues, and whether the metastatic process is driven by events occurring within the primary tumor bulk or if metastases diverge early during tumorigenesis from a primary tumor subclone and evolve separately and in parallel. A better understanding of breast tumor evolution in the metastatic process should lead to new avenues in the diagnosis and treatment of metastatic breast cancer.

Several studies have shown that breast cancer metastases can differ from the primary tumors with regard to key biological markers assessed with conventional methods such as immunohistochemistry and *in situ* hybridization [1-3]. For example, a recent meta-analysis encompassing thousands of patient-matched breast tumors found that the primary tumor and the distant metastasis were discordant in 20% of cases for estrogen receptor status, 33% for progesterone receptor status, and 8% for HER2 status [4]. Moreover, conventional methods such as Sanger sequencing and IHC have shown that, for instance, *PIK3CA* mutations and PTEN status can also change between the primary tumor and matched metastasis [5, 6].

The rapid advancement of next-generation sequencing technologies allows for more comprehensive characterizations of tumor genomes, including not only sequence mutations but also genome rearrangements [7]. So far, genomic studies of paired primary and metastatic breast tumors have been limited to only a few patients or to targeted exome sequencing and the results of these studies diverge in their conclusions: some indicate a large gain in genomic changes, particularly point mutations, after metastasis [8] while other reports suggest that there are more similarities than differences [9-11].

Chromosomal rearrangements can be driver events in tumorigenesis, for example in a number of hematological malignancies and sarcomas [12]. However, rearrangements have not been thoroughly studied in breast cancer, and evidence thus far suggests that they are common and apparently stochastic events with very few rearrangements having been described as recurrent [7]. In the early stages of tumorigenesis, extended proliferation is thought to lead to telomere attrition and dysfunction, resulting in chromosomal rearrangements from breakage-fusion-bridge cycles [13]. Single catastrophic shattering of chromosomes and subsequent aberrant repair can also occur, a phenomenon first described by Stephens *et al*. as chromotripsis [14]. This type of catastrophic event has since been complemented by other complex rearrangement phenomenon such as chromoanasynthesis and chromoplexy (reviewed in [15]). Although chromothripsis has been detected in some breast cancers [16], the frequency of these events, the contribution of alternative mechanisms for generating structural rearrangements, and whether some or all of these are early events or continuously occurring during the evolution of the cancer into metastatic disease, is still not known. Regardless of whether chromosomal rearrangements can be primary drivers of breast tumorigenesis or represent innocuous passenger events, the rearrangements themselves can serve as important biomarkers of the disease (i.e. “fingerprints”) that may be clinically useful, for example in patient monitoring by analysis of circulating tumor DNA [17, 18]. To date, no detailed comparison of chromosomal rearrangements between primary and metastatic breast tumors has been described.

The aim of our study was to characterize the pattern of genomic rearrangements in the primary tumors and distant metastases of eleven breast cancer patients and investigate the level of similarity and clonality between the paired samples.

## RESULTS

### Whole genome sequencing of paired primaries and metastases

**Figure 1:**
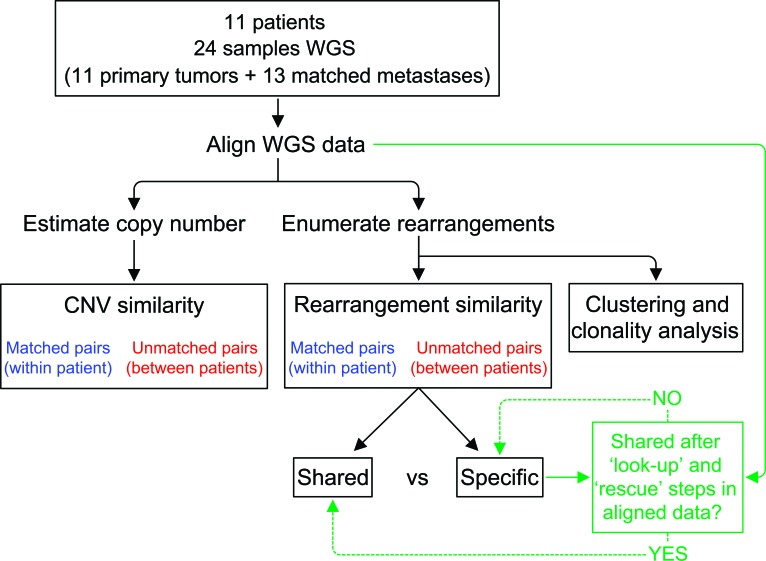
Analysis schema for comparison of structural genomic variation among primary-metastasis breast tumor pairs DNA from fresh frozen tissue from primary and distant metastases from 11 patients were subject to whole genome sequencing (WGS). Estimation of DNA copy number variation (CNV) and identification of chromosomal rearrangements were performed using the aligned WGS data. Copy number similarities and rearrangement similarities were computed as percentages for true pairs (blue) and all possible non-matching pairs (red). As indicated in green, additional “look-up” and “rescue” steps were performed to identify reads supporting a rearrangement and classify rearrangements as shared between pairs or specific to one tumor (see Methods and Supplementary Methods).

Whole genome sequencing (WGS) was performed with a strategy that combined low sequence coverage paired-end sequencing with larger fragment sizes for improved physical coverage (Figure 1). The primary tumor and metastasis of patient 16 (P16) was sequenced to high coverage (67-70X physical coverage corresponding to 34-35X sequence coverage), and *in silico* down-sampling experiments indicated 20X physical coverage to be sufficient to reliably detect most rearrangements. Therefore, we sequenced the remaining tumor samples to a median physical coverage of 22.3X (range 19.3-30.6X) corresponding to a median sequence coverage of 9.3X (range 7.54-14.9X; Supplementary Table S1).

### Identification and pair-wise comparisons of copy number variation

DNA copy number (CN) across the genome was estimated from the WGS data. Focusing on CN events (seen as events regardless of size) in genomic regions with aberrant CN and ignoring regions with normal CN coincident in both tumors, we compared all possible pairings between tumor samples. For all possible tumor comparisons between patients, 5-28% of CN aberrations (CNAs) were shared (median 13%). Primary tumors and metastases from the same patient shared between 16-44% of their abnormal CNAs (median: 35%; Table 2). Notably, some matched tumor pairs were less similar than randomly paired tumors, as the minimum similarity between matched tumors (16%) was well below the maximum similarity (28%) for the unmatched samples, and not much higher than the median value for the unmatched sample pairs (13%). We also performed this analysis taking into account the length of the CN events (i.e. large stretches of deletions and gains will contribute to increase in similarity) as well as shared normal CN areas. As anticipated, the degree of similarity between true paired tumors increased substantially (median 89%, range 42-99%), however this was accompanied by an increase in similarity for random unmatched pairs (median 60%, range 19-98%) with a considerable overlap in percent-similarities to that of true paired tumors (Supplementary Table S1).

**Table 1:** Clinical characteristics for the 11 breast cancer patients

Patient ID	Age at diagnosis	Tumor sample	Time after prior sample (years)	Recurrence Site	ER	PR	HER2	Adjuvant treatment	Survival after diagnosis (years)
P3	68	P			Negative	Negative	Amplified	None	2.5
		M1	1.2	Lung	NA	NA	Amplified		
		M2	0.5	Ipsi breast	Negative	Negative	Amplified		
P6	66	P	0.02		Positive	Negative	Normal	Tam	4.5
		M		Skin	NA	NA	Normal		
P7	54	P			Positive	Negative	Normal	Tam	13
		M	10.4	Skeleton	Negative	Negative	Normal		
P8	53	P			Positive	Positive	Normal	None	2.9
		M	2.5	Greater omentum	Positive	Negative	Normal		
P12	53	P			Positive	Positive	Normal	RT, Tam	13
		M1	5.6	Skin	Positive	Positive	Normal		
		M2		Skin	Positive	Positive	Normal		
P14	44	P			Positive	Positive	Normal	RT, Tam	5.0
		M	4.8	Contralateral axilla	Positive	Positive	Normal		
P15	43	P			Negative	Negative	Amplified	None	7.6
		M	7.1	Skeleton	Negative	Negative	Amplified		
P16	36	P			Negative	Positive	Amplified	None	16
		M	0.6	Contralateral axilla	Negative	Negative	Amplified		
P17	50	P			Positive	Positive	Normal	None	4.9
		M	3.3	Greater omentum	Positive	Negative	Normal		
P18	45	P			Negative	Negative	Normal	None	5.6
		M	3.3	Lung	Negative	Negative	Normal		
P20	58	P			Positive	Negative	Normal	RT, CMF	12
		M	11.2	Lung	Positive	Positive	Amplified*		

Estrogen receptor (ER) and progesterone receptor (PR) were determined per clinical routine, for all patients but the metastasis of P20, using enzyme-immuno assay and cut-off for positivity was 25 fmol/mg protein. For P20 metastasis ER and PR were determined by immunohistochemistry (cut-off >10% positive cells). Human epidermal growth factor 2 (HER2) status was determined from DNA copy number analysis using whole genome sequencing data (cut-off 0.7 log2 ratio). *corroborated by clinical HER2 IHC data (Herceptest score 3+). P, primary tumor; M, metastasis; M1, first metastasis when more than one; M2, second metastasis; Ipsi, ipsilateral; RT, radiotherapy; Tam, tamoxifen; CMF, cyclophosphamide/methotrexate/5-Fluorouracil; NA, not available.

**Table 2:** Chromosomal rearrangement and copy number data for 11 breast cancer patients

Samples			Number of rearrangements			Similarity	
Patient	Sample	Paired sample	Total	Shared	Specific	Percentage of shared rearrangements	Percentage of shared copy number events
P3	P	M1	216	212	4	98%	44%
	M1	P	224	212	12	95%	
	P	M2	202	195	7	97%	40%
	M2	P	212	195	17	92%	
	M1	M2	224	217	7	97%	43%
	M2	M1	229	217	12	95%	
P6	P	M	163	162	1	99%	42%
	M	P	165	162	3	98%	
P7	P	M	78	62	16	79%	16%
	M	P	82	62	20	76%	
P8	P	M	52	36	16	69%	25%
	M	P	59	36	23	61%	
P12	P	M1	38	29	9	76%	28%
	M1	P	43	29	14	67%	
	P	M2	40	30	10	75%	29%
	M2	P	39	30	9	77%	
	M1	M2	38	35	3	92%	36%
	M2	M1	39	35	4	90%	
P14	P	M	54	50	4	93%	33%
	M	P	56	50	6	89%	
P15	P	M	26	16	10	62%	27%
	M	P	18	16	2	89%	
P16	P	M	100	99	1	99%	24%
	M	P	117	99	18	85%	
P17	P	M	82	82	0	100%	40%
	M	P	87	82	5	94%	
P18	P	M	310	269	41	87%	35%
	M	P	404	269	135	67%	
P20	P	M	212	174	38	82%	36%
	M	P	221	174	47	79%	
SUMMARY							
Paired tumors		Median	85	82	10	89%	35%
		Min	18	16	0	61%	16%
		Max	404	269	135	100%	44%
Random pairs		Median	67	3	64	3%	13%
		Min	10	0	8	0%	5%
		Max	367	12	362	36%	28%

P, primary tumor; M, metastasis; M1, first metastasis when more than one; M2, second metastasis.

### Identification of tumor-specific chromosomal rearrangements

Chromosomal rearrangements were evaluated as molecular tumor “fingerprints”. Per tumor sample, a median of 85 rearrangements (range 18 to 404) were identified, in line with prior observations in breast cancer [7]. Primary tumors carried a median of 82 (26-310) rearrangements per tumor compared to distant metastases, which harbored a median of 87 (range 18-404) rearrangements (*p* = 0.026, two-sided Wilcoxon's signed rank test; Supplementary Table S1). The rearrangements comprised a median of 21 interchromosomal fusions (CTX; range 5 to 88) and a median of 70 intrachromosomal events (range 12 to 316), which included intrachromosomal rearrangements (ITX) as well as deletions (DEL) and inversions (INV) (Supplementary Table S1). On average, across our entire sample set, the four different types of rearrangement classes were roughly evenly represented: CTXs comprised an average of 28% (range 11-39% within a tumor) of the total number of rearrangements, ITXs contributed with 23% (range 13-54%) of the rearrangements, DELs with 22% (range 14-32%), and INVs 27% (range 9-41%). The frequencies of these rearrangement types were not significantly different between primary tumors and metastases (*p* > 0.05, Wilcoxon's signed rank test).

### Estimation of similarities of specific chromosomal rearrangements between tumors

We determined the percentage of rearrangements that were shared between tumors or specific to an individual tumor by comparing pairwise the genomic coordinates for both ends of each rearrangement found in one tumor to those in another tumor (see Methods; Figure 1). First we investigated how many rearrangements were shared between tumors from different individuals by performing all possible pairings of tumors (primaries as well as metastases) after excluding the true patient-matched pairs. Between two unrelated samples, only a median of 3% of the rearrangements were shared (range 0-36%; Table 2). In stark contrast, true pairs shared a median of 89% of their rearrangements (range 61-100%; Table 2). In other words, there was a clear separation between the similarity percentages of unmatched and matched pairs (*p* < 2.2×10^−16^, two-sided Wilcoxon's signed rank test).

For all patients but P12 and P15, the rearrangements present in the primary tumor were shared with its paired metastasis in a greater proportion than the rearrangements in the metastasis were shared with its primary tumor. Thus, in 9 of 11 patients, the metastasis generally diverged from its primary tumor, acquiring a greater number of new rearrangements than were lost (Table 2). If a rearrangement was found in the primary tumor there was a very high probability that it would also be found in the metastasis (conditional likelihood P(Met/Prim) = 0.856). In general, a metastasis gained a median of 14 additional rearrangements (range 2 to 135) and lost 10 (range 0 to 41) (Supplementary Table S1).

**Figure 2:**
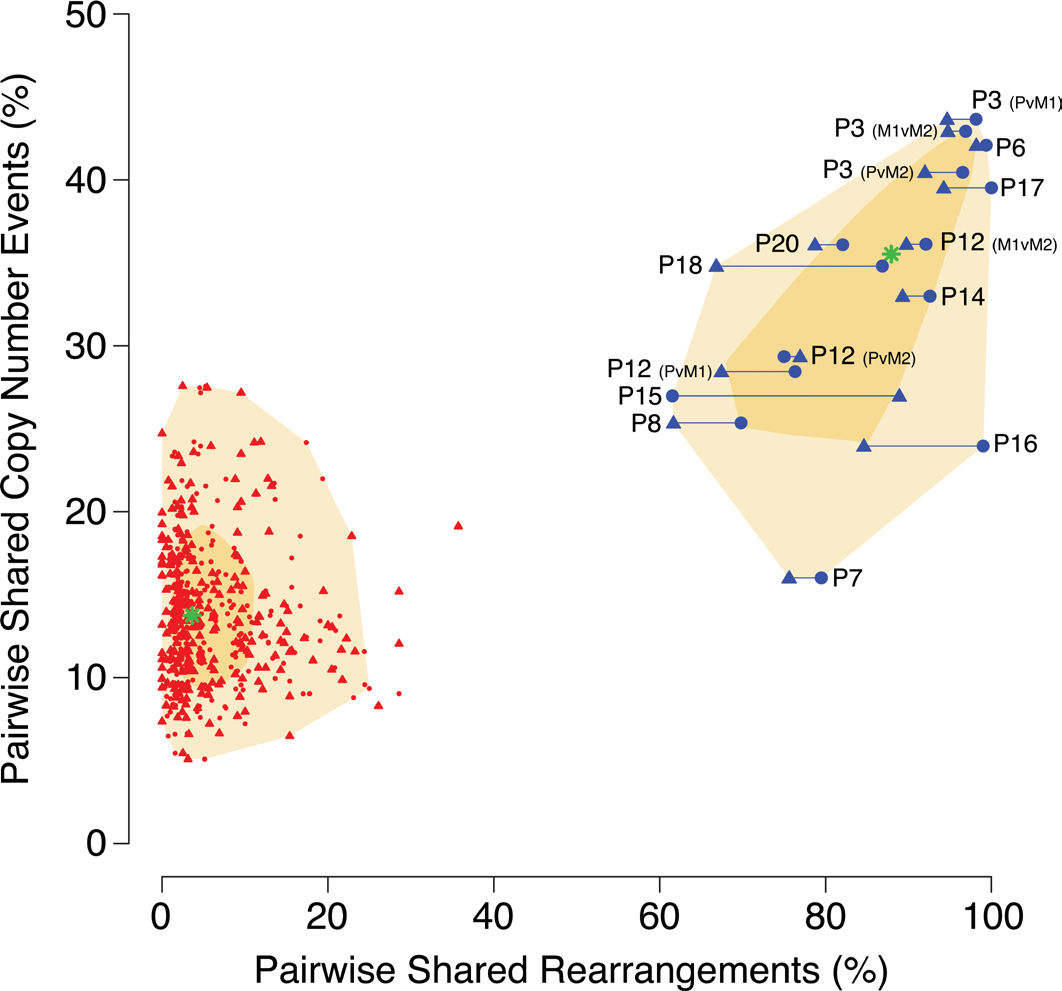
Comparison of tumor similarity based on DNA copy number versus chromosomal rearrangement patterns Similarities between tumor samples are plotted on the basis of their similarity percentages for rearrangements (X-axis) and copy number (Y-axis). For chromosomal rearrangements, paired sample comparisons were made in both directions, generating a percentage value per each tumor in the comparison and thus each pair is represented by two plotted datapoints. True within-patient pairs are plotted in blue and connected with a horizontal line, and all possible unmatched pairings are plotted in red (connecting horizontal lines omitted for greater legibility). The chronologically first event in a pair (primary tumor, P, or first metastasis, M1) is represented by a circle while the chronologically later event (M1, or second metastasis M2) is represented by a triangle. The median value for the respective types of pairings are indicated by a green star and the shadowed areas denotes the 50th (dark yellow) and 95th (light yellow) percentiles.

Patterns of similarity between primary and metastasis tumor pairs were visualized by plotting similarity by chromosomal rearrangements *versus* the similarity by CN aberrations (Figure 2). As can be seen in this figure, in contrast to CNAs which indicated true primary-metastasis pairs to have a similarity in the 16-43% range and random pairs in the 5-28% range, the use of chromosomal rearrangements as similarity markers distinctly separated the patient-matched (61-100%) and unmatched (0-36%) tumor pairs.

### PCR-validation of chromosomal rearrangements in paired tumors

Rearrangements selected from all patients were PCR validated using matched normal DNA, if available, or an unmatched pool of 47 Swedish normal blood DNA samples (see Supplementary Methods) as controls. Of 113 informative assays, 62/62 (100%) of the rearrangements predicted as shared between tumor pairs were validated as shared, and 8/62 (13%) of these were also identified in normal germline DNA (matched normal DNA for 2 cases, or in the normal pool for 6 cases) and thus represent a possible overestimation of tumor-specific shared percentages (Supplementary Table S2). Of 51 rearrangements predicted to be specific to one tumor in a pair, 41 (80%) were validated as specific, whereas 10 (20%) were identified as in fact shared between the matched samples (and not present in the normal controls) and thus represent a possible underestimation of shared percentage. We conclude that our estimates of shared and specific rearrangements have an overall good accuracy with 91% of rearrangements correctly validated (when excluding the 8 germline results).

### Chromosomal rearrangements as tumor-specific barcodes

**Figure 3:**
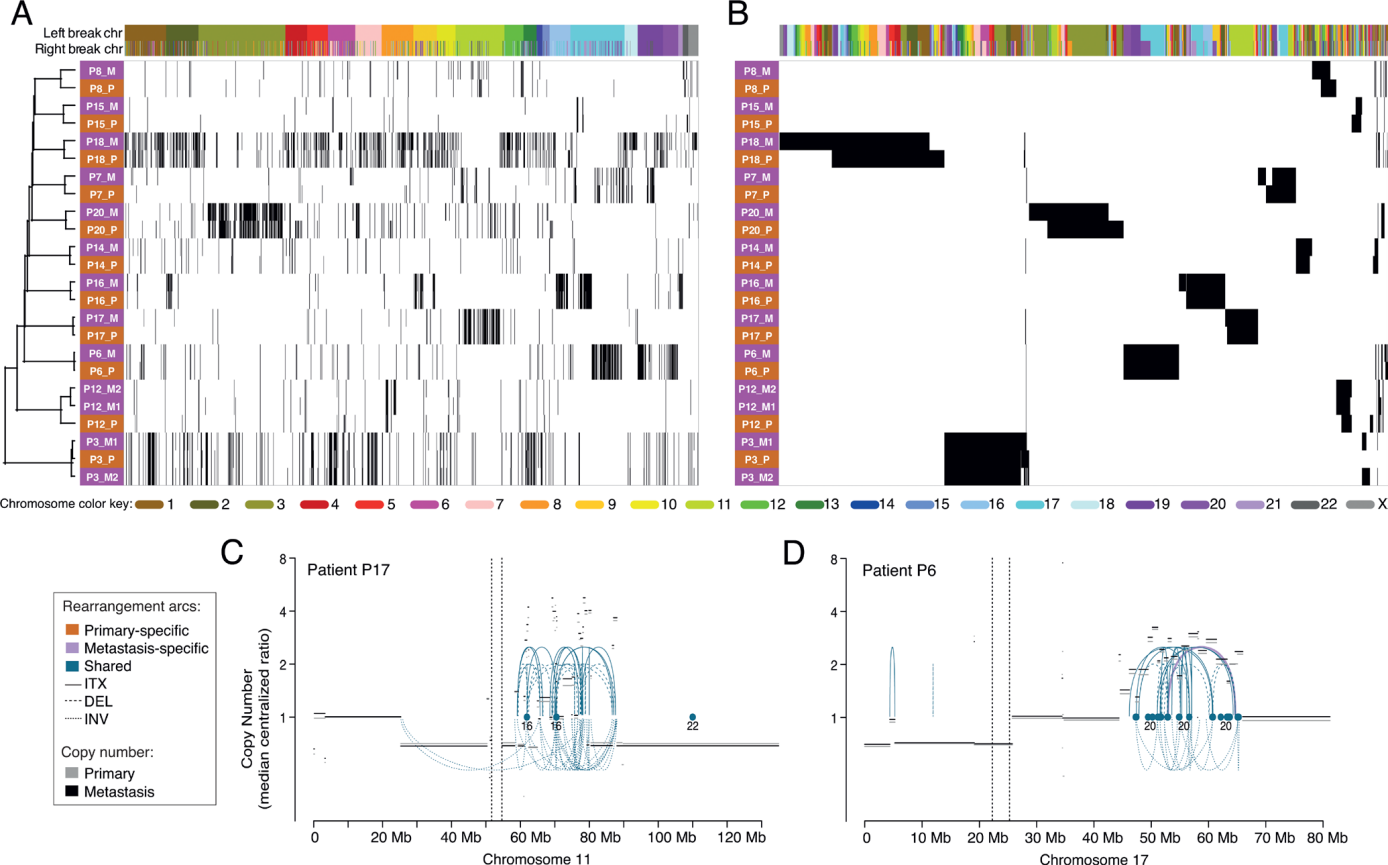
Hierarchical clustering of primary and metastatic breast tumors using all enumerated chromosomal rearrangements **A.**, Clustering of the tumor samples (rows) with the rearrangements (columns) ordered by the genomic location of each side of the rearrangement. **B.**, Two-way clustering where both the rearrangements and the tumor samples were clustered. Primaries are denoted by pink and metastases by orange. Color labels indicate the chromosome for the left and right breakpoints of each rearrangement. Example of clusters of rearrangements and copy number changes in the primary and the metastasis on chromosome 11 of patient P17 **C.** and chromosome 17 of P6 **D.** Arcs represent intrachromosomal rearrangements (ITX), inversions (INV) or deletions (DEL) and the color of the arcs indicate if the rearrangements were found in both tumors (shared) or specific to one of them. No primary specific rearrangements were present in either of the hotspots depicted. Dots indicate rearrangements to another chromosome (CTX) and the numbers below indicate to which chromosome(s).

Similar patterns of chromosomal rearrangements between paired tumors can be further illustrated in a hierarchical clustering of all samples using all non-redundant rearrangements (Supplementary Methods) either sorted according to their locations in the genome or clustered. Samples clustered according to their rearrangement patterns and all primary tumors clustered tightly together with their respective metastases (Figure 3). The clustering illustrates that genomic events were highly specific to clonally related tumors, forming unique “barcode”-like patterns (Figure 3A) and patient-specific rearrangement “blocks” (Figure 3B). All metastases were highly similar to their paired primary tumors, and this appeared to apply regardless of the site of metastasis, multiple metastases, or the time between events (e.g., P3 with a lung metastasis and a later ipsilateral breast recurrence and P12 with two skin metastases 5.6 years after the primary) (Figure 3A, Tables 1 and 2). Even though the P3 metastases occurred in distinct tissues, they displayed remarkable similarities with regard to rearrangements: 97% of the rearrangements identified in the first metastasis in the lung were also found in the second metastasis (in the breast), 6 months later, and 98% of the rearrangements in the second metastasis were present in the first metastasis. For patient P12, the similarity percentages were slightly lower between the two coincident skin metastases (92% and 90%, shared rearrangements respectively).

### Types and patterns of structural rearrangements in the breast cancer genomes

The different tumor genomes displayed diverse patterns of rearrangements and copy number changes (Figure 3A, Supplementary Figure S1). Several genomes were less eventful with a small number of events spread across different chromosomes. Among the tumors carrying large numbers of rearrangements we found distinct patient profiles: some tumors had events distributed more evenly across the entire genome and involving most chromosomes (e.g., P18, notably the only triple-negative tumor pair), while in other highly rearranged tumor genomes the majority of the breakpoints were concentrated to a smaller number of chromosomal regions (e.g., P16) (Figure 3A, Supplementary Figure S1).

The genomic profiles may represent distinct types of complex rearrangements and CN aberrant regions. Some rearrangements appeared to have been isolated events without any other nearby rearrangements. However, it was common to see five, ten or many more rearrangements clustered together in a smaller area of a chromosome arm (as small as 10-20 Mb) or a larger area extending up to the whole chromosome arm, often coinciding with CN changes (Figure 3A, Supplementary Figure S1). In the great majority of these rearrangement-dense foci, most of the rearrangements were shared between the primary tumor and its matched metastasis (Figure 3A, Supplementary Figure S1). Interestingly, the majority of the dense rearrangement clusters were prominently interchromosomal in nature with varying contributions of intrachromosomal rearrangements. Most commonly, the clusters were coincident with either chaotically oscillating CN variations or whole-arm losses/gains, or both. Examples of rearrangement clusters include chromosome 3 of patient P20, on chromosomes 16 and 17 in P16, on chromosomes 17 and 19 in P6, and on chromosome 11 in the tumors of P17 (Figure 3A, 3C-3D, Supplementary Figure S1). Interestingly, most rearrangement clusters originated in the primary tumor and were retained in their metastasis, even in P20 where the metastasis was diagnosed 11 years after the primary tumor. However, occasionally rearrangement clusters were lost in the metastasis (e.g. P20 chromosome 4), whereas others appeared to have added additional rearrangements to the cluster (e.g., P20 chromosome 3 and P8 chromosome 11-22) (Supplementary Figure S1).

### Assessing tumor chromosomal rearrangement clonality

**Figure 4:**
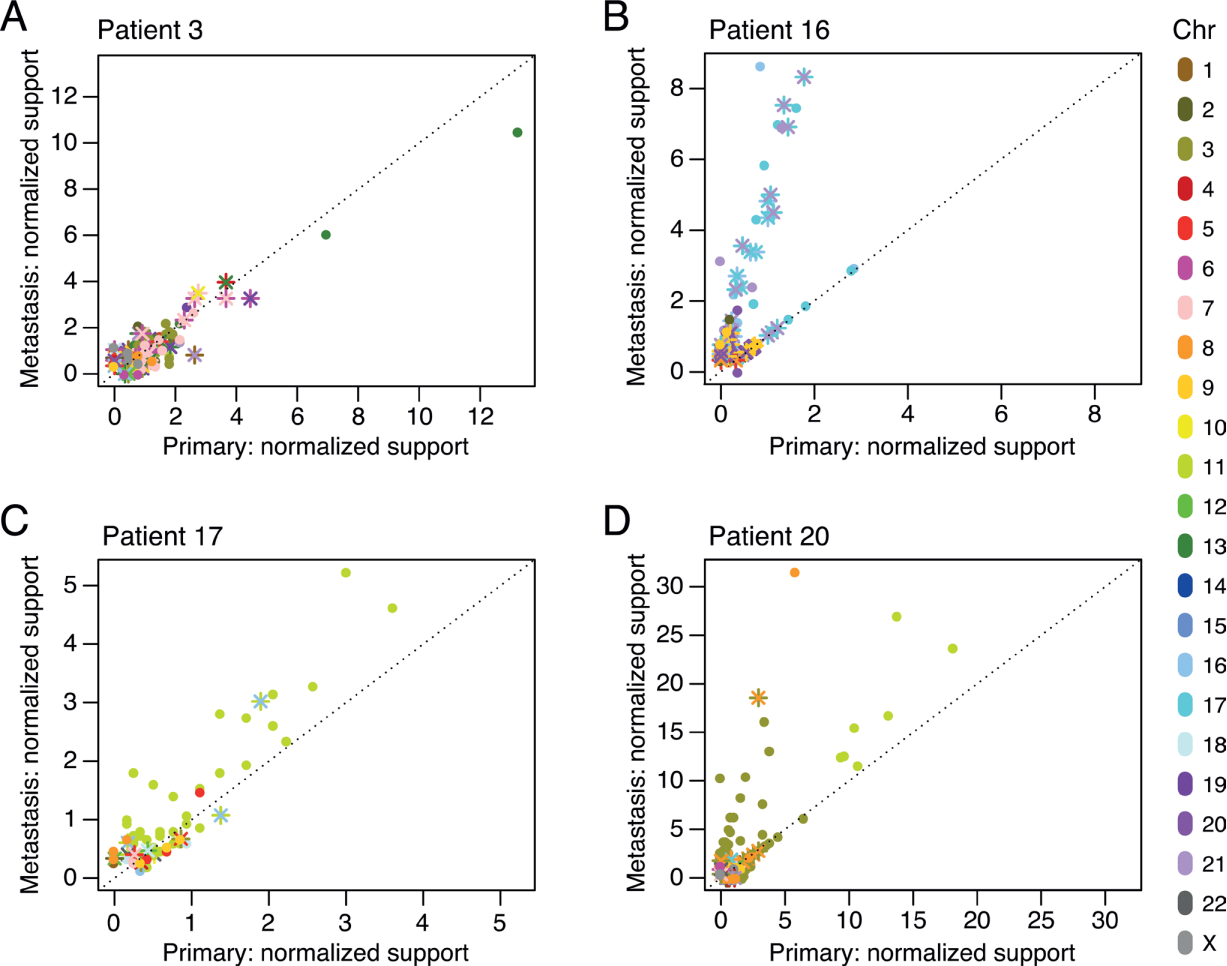
Chromosomal rearrangement clonality plots for patients P3 **A.**, P16 **B.**, P17 **C.**, and P20 **D.** The number of reads supporting each rearrangement, normalized to the overall sample genomic coverage, was plotted for all shared rearrangement events in the primary tumor and the metastasis. Intrachromosomal breakpoints are represented by circles. Interchromosomal rearrangements are represented by two different markers (“x”, “+”) for the two breakpoint sides of the rearrangement. Color labels indicate the chromosome(s) involved in the rearrangement.

The bulk of the rearrangements were shared between samples from the same patient, but throughout the data set, we found that some of them were less pervasive in the primary tumor than in the metastasis or vice versa. To illustrate the variation in clonal fractions of each rearrangement in paired tumors, we plotted each rearrangement according to the read support in the primary tumor and the metastasis after normalization to overall sample sequencing coverage (Figure 4). Shared breakpoints equally represented in each of the paired samples lined up along the main diagonal, as exemplified in patient P3 (Figure 4A). For patients P16, P17, and P20, a portion of the breakpoints lined up along a second diagonal with a steeper slope (Figure 4B-4D), indicating that for these cases a specific set of rearrangements had become supported by a considerably larger number of reads in the metastases. These clonally abundant events in the metastases included a specific subset of rearrangements involving mainly chromosomes 17, 18, and 21 for patient P16, chromosome 11 for patient P17, and chromosomes 3 and 11 for patient P20 (Figure 4). This is consistent with either amplification occurring in these genomic areas or an increase in the cellular representation of a subclone carrying these rearrangements.

### Global hotspot regions for chromosomal rearrangements

**Figure 5:**
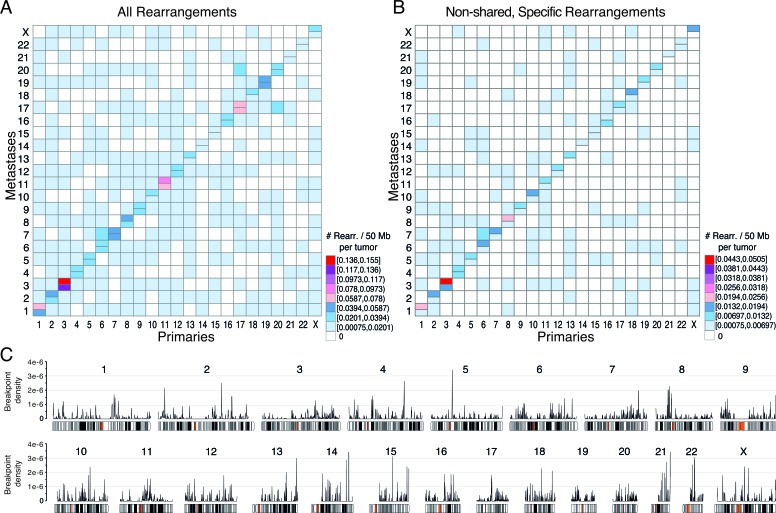
Overview of chromosomal rearrangement hotspots across all breast cancer samples For all rearrangements **A.**, or for only the non-shared rearrangements specific to the primary tumor or to the metastasis in patient-matched pairwise comparisons **B.**, heatmaps of the number of rearrangements per chromosome per 50 Mb illustrate hotspot chromosome combinations in primaries (lower right half) and metastases (upper left half). In A and B, the diagonal indicates intrachromosomal rearrangements with metastasis above and primaries below the horizontal divide. **C.**, Genome-wide breakpoint densities are plotted above chromosome ideograms showing the distribution of rearrangement hotspots along the genome. The genomic coordinates and gene annotations for hotspots with a density >3×10^−6^ are provided in Supplementary Table S4.

As can be seen in Figure 5A, all chromosomes were involved in intrachromosomal rearrangements, occurring at the highest density within chromosomes 3, 11, and 17. Generally, larger chromosomes were more promiscuous in interchromosomal rearrangements, with chromosomes 1 being involved in rearrangements with 18 other chromosomes, and chromosomes 2 and 6 both participating in rearrangements with 17 other chromosomes. Chromosome 21 participated in the fewest rearrangements with other chromosomes (only 6), chromosome 14 the second fewest (7), and chromosome 4 stood out, given its large size, as only rearranging with 11 other chromosomes. Metastases had a slight increase in specific intrachromosomal rearrangements as compared to primaries, in particular with gain of intrachromosomal events in the metastases for chromosomes 3, 8, 13, and X (Figure 5B). Furthermore, global rearrangement breakpoint density plots along each chromosome indicated notable local hotspots at 5p, 13q, 14q, 15q, 21q, and 22q (Figure 5C). The genomic coordinates and the genes within the regions of these rearrangement hotspots (breakpoint density > 3×10^−6^) can be found in Supplementary Table S4.

### Gene annotations for locations of chromosomal rearrangements

Finally, we investigated whether there were breakpoints located in genic areas (−2k promoter to 3′UTR) that potentially could contribute to tumor development and metastasis. Across the whole dataset, we identified in total 3838 rearrangements, thus comprising 7676 breakpoints of which 45.5% (3496) were located in genic areas. To evaluate if there was an enrichment for genic locations we simulated 7676 random breakpoints 10,000 times and found that a median of 43.3% (3323; range 3167-3507) of the random breakpoints were genic (*p* = 0.0002; Supplementary Methods), indicating that there was a marginal but significant 2% enrichment over random for breakpoints to occur in genic regions. Many of the 3838 rearrangements were non-unique since they were shared between tumors, and therefore reduced to non-overlapping 1538 rearrangements (3076 breakpoints). Among the 1538 non-redundant rearrangements, 1327 rearrangements had at least one of their two breakpoints in 985 genic regions (Supplementary Table S3). The most frequently affected gene was *TTC28* with 6 unique rearrangements in 4 patients (Supplementary Table S3; we exclude a 7^th^ unique rearrangement, TTC28-GABRA4, found in a 5^th^ patient as it likely represents a germline structural polymorphism, as shown in [19]). Forty-six of the 985 genes affected by rearrangements (4.7%) are known to be cancer-associated (found in the COSMIC Cancer Gene Census [20]). To investigate if cancer-associated genes were more frequently affected by rearrangements, we simulated 10,000 random draws of 985 genes and found a median occurrence of 26 COSMIC genes (2.6%) with only two draws of 46 or more hits (*p* = 0.0004, two-sided; see Supplementary Methods). Therefore, while the majority of rearrangements in any given tumor is likely to be stochastic, there was a 1.8-fold enrichment over random for tumor-associated rearrangements to affect known cancer genes. Of the affected 46 cancer-associated genes in our dataset, 25 (54%) are reported to be involved in gene fusions (Cancer Gene Census v71 [20]) as compared to 64% (349/547) of the total number of genes in the Cancer Gene Census. Cancer-associated genes such as *TP53*, *ATM*, *RB1*, *PTEN*, *ESR1*, and *RARA* were affected by rearrangements in our dataset (Supplementary Table S3). For example, patient P20, with an ER+ and PR- primary tumor acquired in the metastasis a deletion of exons 5-7 in the *ESR1* gene, and there were rearrangements of the retinoic acid receptor gene (*RARA*) in the HER2-amplified tumors of patient P16.

## DISCUSSION

We have performed the largest whole-genome characterization of chromosomal rearrangements in primary breast tumors and patient-matched metastases to date, and we show that breast cancer metastases share a majority of their chromosomal rearrangements with the initiating primary tumor. The patterns of rearrangements across the genomes were exquisitely patient-specific and essentially non-overlapping between patients. Moreover, similarities within a patient appear to be irrespective of metastatic site, adjuvant therapy, or time between events as metastases arising up to eleven years after the primary tumors still exhibited remarkably similar patterns of chromosomal rearrangement “barcodes”. As discussed below, our results are most consistent with chromosomal rearrangements occurring relatively early in cancer progression, prior to metastatic dissemination, and the majority of rearrangements being maintained during metastatic progression.

Traditionally, cancer has been viewed as a stepwise progressive disease, with genomic and epigenomic aberrations evolving gradually to generate an increasingly malignant disease, often over long periods of time. This view has been challenged by evidence of catastrophic events such as chromothripsis that cause a large number of simultaneous chromosomal rearrangements [14]. In chromothripsis, a one-time cellular crisis event is thought to cause chromosomes to shatter and be re-assembled by the cell's DNA repair machinery creating highly localized areas of rearrangements within or between chromosomes [15]. In chronic lymphocytic leukemia chromothripsis was not ongoing but rather an early event that remained stable during the course of the disease [14]. Similarly, in colorectal cancer, chromothripsis can occur early and precede dissemination of the tumor [21]. Several of the tumor genomes in our study displayed dense clusters of rearrangements in discrete locations of the genome, indicative of chromothripsis, and almost all of these were present already in the primary tumor. Additionally, alternative mechanisms for catastrophic genomic events have been proposed, such as template-switching and other replication-based mechanisms (reviewed in [15]). On the other hand, not all tumors in our dataset carried the highly localized rearrangement clusters. For example, the only triple-negative patient (P18) carried a large number of rearrangements distributed fairly randomly across the genome. Regardless of the rearrangement pattern, the high rate of shared rearrangements between primary and metastasis is consistent with their early origin, potentially during a time of telomere crisis, where high proliferation already during ductal hyperplasia and carcinoma *in situ* leads to telomere erosion, chromosome fusing, break-fusion-bridge cycles, and complex chromosomal rearrangements [13, 22]. Interestingly, the majority of rearrangements found in primaries were also found in its metastasis, indicating that the rearrangements did not appear to be under significant negative selection and may even be maintained under positive selection. Furthermore, multiple metastases were nearly identical (shared > 90% of rearrangements) for two patients, P3 and P12, consistent with their seeding by a common parental clone without much subsequent chromosomal instability.

Chromosomal rearrangements have been suggested to promote cellular transformation by activation of oncogenes or loss of tumor suppressors [14, 23]. We found that breakpoints occurred more often in genic regions than was expected by chance, consistent with the literature [7]. Moreover, within the group of affected genes there was a 1.8-fold enrichment for known cancer-associated genes. This could suggest that, although many chromosomal rearrangements appear to be early tumorigenic events and stable through tumor progression, they are not completely stochastic. Alternatively, stochastic rearrangements undergo selection, leading to cancer gene enrichment. One may also speculate that these findings could be related to some features of chromatin, such as open, gene-dense, and actively transcribed euchromatin, or to local sequence contexts such as interspersed nuclear elements and other repeats [7].

The identified genomic rearrangements affected genic areas of a number of known cancer driver genes, *e.g.*, *TP53, ERBB2, EZH2, ATM*, *RB1,* and *PTEN* but a large number of affected genes were not previously associated with cancer (not found in COSMIC) and further studies are needed to explore whether these could constitute new cancer-associated genes. To predict the functional effects of the often complex rearrangements in the vicinity of known genes, particularly for oncogenes, further characterization is required. The most frequently rearranged gene, *TTC28*, which hosts an L1 retrotransposition element, has previously been shown to be commonly rearranged in colorectal cancers but whether these rearrangements are functionally relevant to cancer remain unclear [19, 24]. Rearrangements of the retinoic acid receptor gene (*RARA*) were found in the HER2-amplified tumors of patient P16. The association between *RARA* rearrangements and HER2-amplified breast cancer was also seen in a recent study [10]. The differences we observed between paired tumors were primarily characterized by a modest accumulation of new rearrangement events in the metastasis. Some of the new chromosomal rearrangements appearing exclusively in the metastases were found within known cancer genes such as *PTEN, FHIT*, and *ESR1*. For example, the patient P20 metastasis-acquired deletion of exons 5-7 in *ESR1,* which removes the ligand-binding domain, may result in an increased ER-signaling: both primary and metastasis were ER-positive; however PR, suggested as a surrogate marker for an activated ER-pathway [25], switched from negative in the primary to strongly positive in the metastasis. Whether this and other genomic rearrangements occurring exclusively in metastases can contribute to an increased metastatic ability or therapy resistance requires further study. Conversion of clinical receptor status of ER, PR, or HER2 during breast cancer progression is not uncommon [26], may be the result of selection pressures, and was evident for several patients in our study (P7, P8, P16, P17, P20). The mechanisms of receptor conversion are not well understood but it appears that, despite an overall considerable concordance in structural variation between paired primaries and metastases, receptor conversions can occur and may be due to genomic rearrangements affecting the receptor gene itself. However, other factors such as epigenetic changes, point mutations and indels, copy number alterations, or other factors may also contribute to receptor conversion.

Our approach of using “rescue” and “look-up” curation strategies to address less prominently supported rearrangements in a matching tumor allowed us to also identify rearrangements that were present only in a smaller subclone in the matched tumor. Indeed, in our PCR-based validation, we had no false-positive results for any of the rearrangements shared between paired tumors - all breakpoints that were considered shared in our analysis of the WGS data were also confirmed present in both tumors during validation. Only 9% of all the PCR-validated rearrangements were misclassified with respect to shared or tumor-specific calls (excluding germline) and all of these were events that had been categorized as tumor-specific in our sequencing data but found to be shared by PCR. Hence, at this sequencing coverage and with our analysis strategy, very few shared rearrangements were underdetected due to clonality and low representation within the tumor bulk.

Our study has a number of limitations. We were limited in the number of patients analyzed and by the metastatic sites that were sampled clinically. We also did not have available normal DNA for all patients and instead relied on a pool of normal controls. As a consequence, some of the identified rearrangements may be patient-specific germline events and hence constitute false-positive events. However, there was no trend that the degree of similarity between paired tumors for the three patients where matched normal DNA was sequenced was lower than for the patients without matched normal DNA, indicating that this false-positivity was limited. In addition, using the same approach, also lacking matched normal DNA in many cases, we previously found that two independent contralateral primary tumors from the same patient shared comparably very few rearrangements, suggesting the false-positive germline contribution to be limited [27]. Together with the results from our PCR validation these data suggest that our method detects primarily somatic events. Despite the limitations of this study, it is the largest paired analysis of primary and metastatic breast cancers by WGS, and our pipeline had high accuracy as determined by PCR validation. Compared to the rearrangements, CN alterations were less informative for determining clonal relatedness. CN data is more complex to compare, there are intrinsic subtype-associated CN alterations [28], issues of CN thresholding and noise, and CN profiles can be strongly influenced by the degree of normal cell content in grossly-dissected tumor specimens [29, 30]. Regardless of approach, whether focusing on CN events as discrete events or when considering genomic length and including regions of shared normal CN, neither CN similarity metric showed a clear separation of true pairs from random pairs. Therefore, CNV profiles was not a useful way to study clonality and we conclude that comparisons of chromosomal rearrangements are more informative. Further studies with more samples, deeper sequencing coverage, and using matched normal DNA will be needed to further characterize the degrees of concordance for both chromosomal rearrangements and in particular CNVs between paired primaries and metastases.

Intratumoral heterogeneity is considered one of the biggest challenges in cancer diagnosis and treatment, where subclones are believed to contribute to the development of treatment resistance. For accurate diagnosis and selection of optimal treatment, it is critical to know whether the tumor specimen analyzed for biomarkers is representative of the whole tumor or whether the phenotype/genotype of the primary tumor is representative of the subsequent metastasis. Our data on chromosomal rearrangements provides evidence that prominent genomic rearrangements are indeed very similar between primaries and matched metastases. Although we have not specifically studied the extent of heterogeneity within our primary tumors and significant heterogeneity may exist, our results suggest that the aggregate heterogeneity of chromosomal rearrangements within a primary tumor largely persists within the distant metastases it seeds. However, the distribution of different subclonal populations may vary slightly between the primary and the metastasis as seen in the increased read-representation for some rearrangements in the metastases of a few patients. Whether these modest changes in genomic rearrangements can contribute to the metastatic process or therapy resistance needs to be explored further. *ESR1* point mutations conferring hormonal therapy resistance, on the other hand, were recently found in breast cancer metastases but not in matching primaries [31-33]. Genomic studies of breast cancers have suggested that the number and repertoire of somatic point mutations vary markedly between different individuals [34, 35] and between cells within the same tumor [36]. Perhaps intratumor heterogeneity as well as the discordance between primaries and metastases is far greater for smaller somatic point mutations than for larger structural genomic changes, indicating that point mutation could be a more common mechanism to promote the metastatic process or develop therapy resistance in breast cancer [37].

Our results suggest that tumor-specific rearrangements could serve as excellent tumor-specific biomarkers in breast cancer, particularly considering their consistency from primary to metastatic disease even over long periods of time. We and others have shown that this could be utilized for *e.g.,* detection of tumor-specific rearrangements in DNA in cell-free blood plasma for ascertaining minimal residual disease, as an early sign of recurrence, or for monitoring therapy response [17, 18, 38, 39]. Chromosomal rearrangements could also be utilized to examine clonal relationships between tumors and determine whether they share a common origin or if they are separate occurrences. We have found this to be useful *e.g.,* in the case of bilateral breast cancer where it is possible to establish if the second tumor for some patients in fact is a metastasis rather than a new primary, which can help guiding the treatment strategy [27]. Finally, our approach may also be highly relevant to other issues in oncology since clonality, tumoral heterogeneity, and DNA biomarkers will likely be important clinical concerns for all cancer types.

## MATERIALS AND METHODS

### Patients and samples

Eleven patients diagnosed with primary breast cancer in the south Swedish healthcare region during 1986-1997 with available frozen specimens from the primary tumor as well as distant metastases arising between 0 and 11.2 years (median 3.3) after initial diagnosis were included in the study (Table 1). For two of the patients, tumor specimens from two different distant metastases were available; therefore in total 24 tumor specimens were studied. Blood samples were available for 3 of the patients (patients 7, 15, and 16) and from 7 unrelated persons and were used as normal controls for whole-genome sequencing. All clinical data except HER2 status was extracted from patient charts (Table 1). The study was approved by the Regional Ethical Review Board of Lund (diary numbers 2009/658, 240-01).

### Whole genome sequencing and analysis

Detailed procedures are described in the Supplementary Methods. In brief, DNA was extracted from fresh frozen tumors using the AllPrep method (Qiagen). Genomic DNA was fragmented to ∼700 bp average fragment size and the TruSeq DNA sample preparation kit was used to generate indexed DNA libraries for sequencing on a HiSeq 2000 instrument (Illumina). The resulting 2×100 bp paired-end reads were aligned to the GRCh37 human reference genome using Novoalign (Novocraft Technologies; Supplementary Methods).

Whole genome sequencing data from the 24 tumor samples and a pooled dataset from 10 normal blood DNA whole genomes was used to perform copy number (CN) analysis (Supplementary Methods). DNA CN was estimated using FREEC [40], and CN variation (CNV) profiles were compared within pairs of samples based on windows delineated by the union of their CNV-segmentation breaks as detailed in Supplementary Methods. The fraction of shared aberrant CNV profiles between two tumors was estimated after excluding all windows with a normal CN state simultaneously in both samples.

Chromosomal rearrangements were enumerated in each tumor sample and a pooled dataset from 10 normal DNA samples using BreakDancer [41] (Supplementary Methods). A catalogue of rearrangements in each tumor and normal sample was created. In the first pass, a tumor rearrangement was kept if it was supported by a number of read-pairs equal to or greater than one-third of the sample's average sequence coverage (i.e., 3 read-pairs if the average sample coverage was 9X). Further filters were applied to the tumor catalogue to remove likely false-positive or germline calls: rearrangements that were detected in the normal sample pool, intrachromosomal rearrangements smaller than 7 kb, centromeric regions, and segmental duplication regions (details in Supplementary Methods).

A chromosomal rearrangement consists of two breakpoints. Using custom scripts, rearrangements were compared between tumors in matched and unmatched patient sample pairs. For each comparison, rearrangements were classified as being “shared” (common to both samples) if the distances of each rearrangement breakpoint matched within a +/− 500 bp window for both sides of the rearrangement. The remaining potential sample-specific rearrangements of a pair were further examined for the presence of sub-threshold matches in the initial BreakDancer calls of the other sample in the comparison (the “rescue” procedure) and reclassified as shared if matches were found. In addition, each remaining sample-specific rearrangement was “looked-up” in the sequence data of the other sample for the presence of covering read-pairs that were missed by BreakDancer (Supplementary Methods and Figure 1). If at least one read-pair supporting both ends of a rearrangement was found in the sequencing data of the other tumor the rearrangement was classified as shared. For each sample, we quantified the number of shared and specific rearrangements and calculated the fraction of shared rearrangements in both directions (*i.e*. (tumor1 overlap with tumor2)/total tumor1, (tumor2 overlap with tumor1)/total tumor2) so that one percentage per tumor was generated (Figure 1). In addition, a combined similarity percentage, based on the union of specific and shared rearrangements found in both samples, was calculated (shared(tumor1 and tumor2)/union (tumor1+tumor2).

### Tumor-specific gene rearrangement “barcodes”, read coverage-based clonality analysis, and rearrangement density analysis

A “genomic barcode” for each patient was built using a contingency matrix summarizing the presence or absence of rearrangements obtained by clustering all non-redundant identified events. Patient-specific rearrangement patterns were identified through hierarchical clustering using binary distance and ward linkage.

The degree of clonality of each chromosomal rearrangement in the different samples was investigated by analyzing the sequencing read support for all identified rearrangements. Clonality plots were generated by normalizing the number of supporting reads for each rearrangement by the overall sequence coverage of the respective sample and plotting the metastasis against the primary tumor.

The occurrence and location of potential hotspots for chromosomal rearrangements in the whole sequencing dataset was investigated in two ways. Firstly, the number of rearrangements per chromosome, from both ends of the rearrangements, and tumor type (primary or metastasis) was plotted in a heatmap. Secondly, an R *density* function with a bandwidth of 10 kb was used to generate a genome wide breakpoint density track above chromosome ideograms for all tumors to show the distribution of hotspots across all chromosomes of the genome. Since rearrangements are two-sided, each rearrangement provided two breakpoints for the calculation of the density.

The genomic rearrangement profile of each tumor was plotted using Circos [42], and the heatmaps were generated using the ggplot2 R package. All other plots were generated using standard R graphical libraries.

### PCR validation of rearrangements

Rearrangements were validated with conventional PCR. Our in-house SplitSeq bioinformatics pipeline was used to retrieve the local sequence around each breakpoint [18]. The rearrangements for validation were randomly selected from all specific rearrangements and all shared rearrangements for each patient. Primers were designed to span the putative breakpoints, generally < 70 bp from each side of the junction, and touchdown PCR was performed on DNA extracted from the primary tumors and metastases (further details in Supplementary Methods). Matched normal DNA was used as a germline control where available (patient 7, 15 and 16). For the others, a normal DNA pool was created from normal lymphocyte DNA extracted from 47 healthy controls.

## SUPPLEMENTARY MATERIAL METHODS FIGURE AND REFERENCES










