# Supplementary material for: Remarkable similarities of chromosomal rearrangements between primary human breast cancers and matched distant metastases as revealed by whole-genome sequencing
Source: Oncotarget. 2015 Oct 2;6(35):37169–84. doi: 10.18632/oncotarget.5951 (PMC4741922; doi:10.18632/oncotarget.5951)
Supplement: Supplementary file 1 [file oncotarget-06-37169-s001.pdf]

Remarkable similarities of chromosomal rearrangements between primary human breast cancers and matched distant metastases as revealed by whole-genome sequencing

Supplementary Material

SUPPLEMENTARY METHODS

DNA extraction, whole genome sequencing and alignment

Fresh frozen tumors were partitioned and homogenized in Buffer RLT using a Qiagen TissueLyser LT (Qiagen) for 8 min at 50 Hz, and the lysate passed through QiaShredder spin columns (Qiagen) according to the manufacturer’s instruction. DNA was extracted using Qiagen Allprep DNA/RNA Mini Kit automated on the Qiacube system (Qiagen). Normal genomic DNA was isolated from blood using the Wizard Genomic DNA Purification method (Promega) according to the manufacturer's instructions. Purified DNA was sheared with Covaris focused-ultrasonication to an average fragment length of 700 bp, and the product analyzed using Agilent BioAnalyzer High Sensitivity DNA assay. One microgram fragmented DNA was used for a library preparation with the TruSeq DNA sample preparation kit (Illumina) according to the manufacturer’s instructions. Before PCR amplification, each library was size-selected for fragments from 550- 950 bp using agarose gels. Libraries were analyzed by BioAnalyzer and the concentrations were measured using a Qubit spectrophotometer (Invitrogen). The barcoded libraries were sequenced on a Illumina HiSeq2000 instrument to generate 2x100 bp reads (in-house and BGI Tech Solutions). Paired-end reads from whole genome sequencing were aligned to the Genome Reference Consortium human reference (GRCh37; SNP patched; with v5 decoy sequences (http://ftp.1000genomes.ebi.ac.uk/vol1/ftp/technical/reference/phase2_reference_assembly_sequence/hs37d5.slides.pdf) using Novoalign v2.07.18 (Novocraft Technologies) with a 2 million read pre-alignment to estimate insert size from the data. Picard Tools v1.66 was used to merge aligned

patient-specific datasets from multiple lanes and to flag duplicate reads.

Copy number estimation from WGS data

Copy number was evaluated from the whole-genome sequencing data using FREEC v6.2 with custom parameters (*breakPointThreshold* 0.95, *forceGCcontentNormalization* 1), in 10 kb windows, and using the pooled data from the 10 normal genomic DNA samples (3 matched patients, P7, P15, P16, and 7 unmatched patients) (1). A custom R algorithm based on an Epanechnikov kernel density estimation was used to re-centralize each copy number profile. A filtering step that removed recurrent regions with abnormal copy number (<0.9 or >1.1 linear ratio and removal done in nearest 10k window steps) in 9 out of the 10 normal samples was applied to each copy number profile.

Copy number profiles of each sample in a pair were compared to one another based on windows defined by the union of their copy number segmentation breaks with an additional 10k window split on each side of each copy number break to increase the resolution at segmental change points.

Each copy number window was assigned a copy number state: +1 (gained, ≥ 1.15 linear ratio), 0 (copy number neutral, between 0.85 and 1.15 linear ratio), and -1 (lost, ≤0.85 linear ratio), and a "slope", corresponding to the difference between the current and preceding copy number linear value. A copy number window with the same state and slope was considered shared.

The degree of similarity of aberrant copy number profiles between two samples was estimated in two ways, either by calculating the fraction of shared windows after excluding all '0' windows with a normal, diploid state simultaneously in both samples (*i.e.* the fraction of shared abnormal copy number events) or by taking into account in the calculation the size of copy number events and all

shared diploid windows (the total fraction of genomic length sharing the same copy number events).

Analysis of chromosomal rearrangements

Enumeration of chromosomal rearrangements and analysis

BreakDancer v1.11 was used to identify chromosomal rearrangements in each sample using default parameters (min 2 supporting read pairs) (2). In addition to the 11 tumor pairs (24 samples, 2 patients with a primary and 2 metastases), rearrangements were identified in 10 normal DNA samples (3 matched, 7 unmatched) and a pooled dataset of rearrangements from all normal samples was created. Per tumor, a rearrangement was kept if it was supported by the number of read-pairs equal to or greater than one-third of the sample's average sequence coverage (rounded to the lowest integer; i.e., 3 read-pairs if the average sample coverage was 9X). Rearrangements ocurring within 1 kb to those identified in the normal pool with 1 or more read-pair support or in each of the 10 normal samples with default 2 or more read-pair support were removed as an initial cleanup of germline events. Furthermore, to reduce alignment noise, additional filtering was performed as follows: rearrangements mapping to centromeric regions (+/-500 kb from the UCSC hg19 *gaps* annotation track), regions of segmental duplication (within 1kb from the UCSC hg19 *genomicSuperDups* annotation track) and small intrachromosomal rearrangements (<7kb) were removed.

To compare tumor pairs, BedTools v2.18 (3) was used to obtain the overlap of candidate rearrangements between samples. Using custom scripts, we compared rearrangements between tumors in matched and unmatched patient sample pairs and, for each comparison, classified rearrangements as being "shared" (common to both samples) if the distances of each

rearrangement breakpoint matched within a +/– 500 bp window for both sides of the rearrangement.

Because rearrangements can be supported by fewer reads in some tumors due to subclonality and because BreakDancer occasionally failed to detect rearrangements in highly rearranged or insufficiently covered genomic regions, each candidate tumor-specific rearrangement was re-examined using two sequentially applied procedures: rearrangement “rescue” and BAM file “look-up”.

The candidate sample-specific rearrangements were further examined for the presence of sub-threshold matches in the initial BreakDancer calls for the other sample in the comparison (the “rescue” procedure). In the rescue procedure, specific rearrangements from one tumor that matched rearrangements in the filtered BreakDancer output of the other tumor but with insufficient read-pair support (less than one-third of the sequence coverage, down to 2 read-pairs), were recovered and the rearrangements reclassified as shared. In the pair-wise “look-up” procedure, specific rearrangements from one tumor were computationally searched for in the aligned sequence file (BAM file) of the other sample and if one or more read-pairs matching within +/– 1kb on each side of the coordinates were detected, the rearrangements were considered shared. Similarly, in addition to the initial clean-up, a germline BAM file “look-up” was also performed against the normal pooled BAM file and rearrangements supported by at least 1 read-pair +/– 1 kb in the normal pool were removed.

For each sample, we quantified the number of shared and specific rearrangements and calculated the fraction of shared rearrangements in both directions (e.g. (tumor1 overlap with tumor2)/total tumor1, (tumor2 overlap with tumor1)/total tumor2) so that one percentage per tumor was generated. In addition, a combined similarity percentage, based on the union of specific and

shared rearrangements found in both samples, was calculated (shared(tumor1 and tumor2)/union(tumor1+tumor2). In addition to the comparisons of chromosomal rearrangements and abnormal copy number events between matched samples, we enumerated all possible combinations of unmatched tumor pairs that could be made out of our 24 tumors (523 patient-unmatched combinations).

Determination of the minimum required sequencing depth per sample

To determine the minimum sequence coverage needed to achieve comprehensive and robust comparisons between paired samples, we performed deep-coverage whole genome sequencing on three samples from one patient in our dataset (patient P16: primary, metastasis and matched normal) at a target sequence coverage of 34X. Sequencing data was aligned as described above. *In silico* down-sampling at [5,10,15,20,25,30,35,40,50,60,70,80,90]% of the total coverage was then performed and rearrangements were identified, requiring a one-third supporting read-threshold with 2 supporting reads as the minimum [2,2,2,2,2,3,3,4,5,6,7,8,9]. The percentage of rearrangements of the master-list (with a one-third sequence coverage, read-support threshold) found in each down-sampling was calculated and used to build a cumulative frequency plot. The minimum optimal sequence coverage threshold was empirically determined to be 20X physical (or 10X sequence) coverage by choosing the value when the fraction of discovered rearrangements exceeded 80%.

Rearrangement barcode and clustering of chromosomal rearrangements

To determine all non-redundant identified rearrangements, we clustered rearrangements according to their genomic coordinates, chromosome by chromosome taking the left side (lower

genomic coordinate) of the rearrangement as reference. After ordering rearrangements by the order of the first left side chromosome, for each left-right chromosome pair, we performed hierarchical clustering using Euclidean distance and single linkage. Clusters were extracted by cutting the obtained tree (R *cutree* function) at a height of h=10000. Using this cutoff we obtained clusters of rearrangements encompassing rearrangements that are not more distant from each other than a median distance of 8 nucleotides (min in both sides 1, max of left side: 9298, max of right side: 3708, mean of left side: 92.8, mean of right side: 85.7). A contingency table recapitulating the presence or absence of each cluster in each sample was built and patient-specific rearrangement patterns were identified by hierarchical clustering. Row-wise clustering was performed using binary distance and ward linkage, and visualized using a customized version of the R *gplots heatmap.3* function (4).

Genome-wide density track of chromosomal rearrangements

A genome-wide breakpoint density track for the rearrangements from the barcode clustering from all samples put together was generated using the R *density* function (bandwidth 10kb) and plotted above chromosome ideograms obtained using the *plotCytoband* function found in the *SNPchip* (5) R package to show the genomic distributions of hotspots in the dataset across the genome. Since rearrangements are two-sided, each rearrangement provided two breakpoints for the calculation of the density.

Rearrangement frequency heatmaps by chromosome

The number of identified rearrangements that occurred on each chromosome was reported in a frequency heatmap for all tumors, and all primary tumors and metastases separately. Since

rearrangements are two-sided, each rearrangement was represented by two breakpoints. The heatmaps were generated by reshaping the table containing the number of breaks per chromosome for a primary and its metastasis using the *reshape2* R package, and plotting them using the *RColorBrewer* and *ggplot2* R packages. The numbers of breaks were normalized to the mean length of the involved chromosomes, and plotted as per 50 Mb per tumor.

Analysis of subclonality using sequencing read support

The clonality plots were generated by normalizing the number of supporting reads for each rearrangement by the overall coverage of the respective sample, and plotting the value of the metastasis against the primary tumor's using base R graphics functions. For intrachromosomal rearrangements, different markers ("x", "+") were used to indicate the two ends of the rearrangements.

Gene annotations for chromosomal rearrangements

We obtained gene annotations for the list of all filtered common and specific rearrangements of all samples using the adjusted coordinate of the fusion breakpoint sequence of each rearrangement obtained with our in-house SplitSeq pipeline (6). Gene annotation for each side of each rearrangement, according to the UCSC hg19 reference, was obtained by running the gene annotation tool (annotatePeaks) provided by the Homer software package (7). We report 'genic' regions defined as from 2kb upstream the promoter through to the 3'UTR (i.e not labelled as 'intergenic' by the annotation script). We used the latest available COSMIC Cancer Gene Census (v71) to check whether rearrangements occurred within previously reported cancer genes (8). To determine whether there was enrichment of breakpoints in genic regions, we generated 7676

(same number as observed) random breakpoints 10,000 times and found that a median of 42% (3240; range 3138-3369) of the random breakpoints were genic. Similarly, to investigate whether known cancer-associated genes (genes in the COSMIC database) were enriched amongst the genes affected by rearrangements we performed 10,000 random draws of 985 genes (same number as observed) from the hg19 RefSeq and calculated the number of genes per draw that were present in the COSMIC database (8). For both enrichment tests, the number of randomizations out of the 10,000 that were equal to or exceeded our observed value were used to calculate two-tailed *p*-values.

Visualization of copy number profiles and chromosomal rearrangements

The genomic profile of each tumor was summarized by a circular diagram drawn with Circos 0.66 (9). Chromosome plots of copy number profiles and chromosomal rearrangements displaying copy number as segments, interchromosomal rearrangements as points (one for each side per CTX) and intrachromosomal rearrangements (ITX, DEL, INV) as arches were drawn using the *draw.ellipse* function of the *plotrix* R package (10). The *bagplot* function (R *aplpack* package (11; http://www.wiwi.uni-bielefeld.de/com/wolf/software/aplpack.html) was used to generate bi-dimensional scatterplots (of copy number vs. chromosomal rearrangement similarities).

Conventional PCR for detection of rearrangements

Rearrangements were validated with conventional PCR. The rearrangements for validation were randomly selected from a list of all specific rearrangements and a list of all shared rearrangements for each patient. Our in-house SplitSeq bioinformatics pipeline was used to automatically retrieve the local sequence around each breakpoint (6). PCR primers were designed to be located on both sides of 198 identified rearrangements so that the amplicon would span the breakpoints (average

amplicon size 144 bp, SD 73). Heavily rearranged areas in the genome proved harder to design PCR primers for, however, for 113 rearrangements the assays were informative and generated an accurately sized PCR-product for the sample where the event was first detected. PCR was performed using a touchdown protocol and input DNA extracted from the primary tumors and metastases. Matched normal DNA was used as a germline control where available (patient P7, P15 and P16). For the others, a normal DNA pool was created from normal lymphocyte DNA extracted from 47 healthy controls.

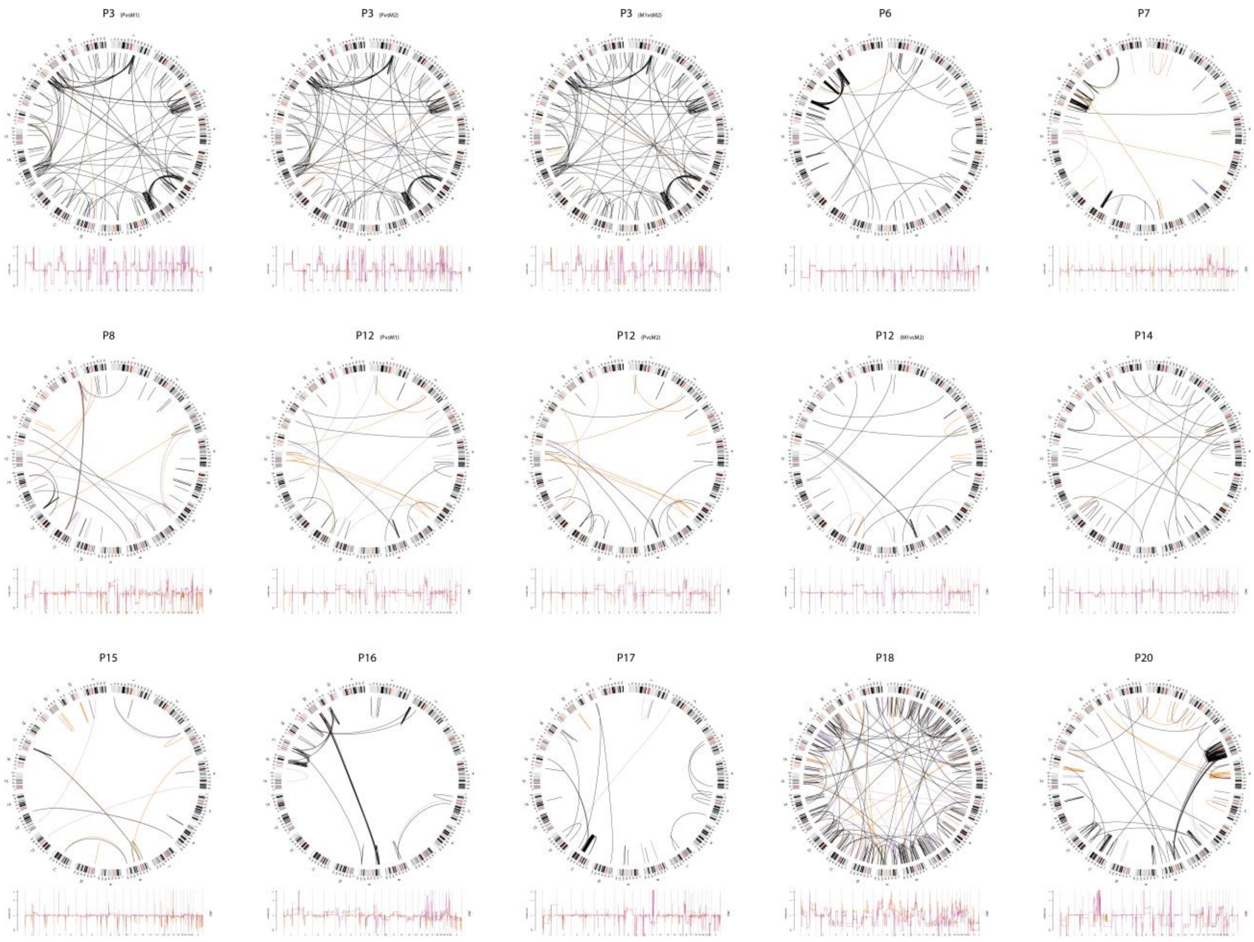

Supplementary Figure S1. Chromosomal rearrangements visualized as Circos plots for all patients. Primary and metastasis from the same patient are overlaid, with orange arches denoting primary-specific rearrangements, purple arches denoting metastasis-specific rearrangements, and black arches denoting the rearrangements that are shared between the two tumors. Below, DNA copy number is plotted in orange for the primary and purple for the metastasis.

REFERENCES

1. Boeva V, Zinovyev A, Bleakley K, Vert J-P, Janoueix-Lerosey I, Delattre O, et al. Control-free calling of copy number alterations in deep-sequencing data using GC-content normalization. Bioinformatics. 2011;27:268–9.

2. Chen K, Wallis JW, McLellan MD, Larson DE, Kalicki JM, Pohl CS, et al. BreakDancer: an algorithm for high-resolution mapping of genomic structural variation. Nat Meth. 2009;6:677–81.

3. Quinlan AR, Hall IM. BEDTools: a flexible suite of utilities for comparing genomic features. Bioinformatics. 2010;26:841–2.

4. heatmap.3 [Internet]. [cited 2014 Oct 16]. Available from: https://gist.github.com/nachocab/3853004

5. Scharpf RB, Ting JC, Pevsner J, Ruczinski I. SNPchip: R classes and methods for SNP array data. Bioinformatics. 2007;23:627–8.

6 Olsson E, Winter C, George A, Chen Y, Howlin J, Tang MH, Dahlgren M, Schulz R, Grabau D, van Westen D, Ferno M, Ingvar C, Rose C, et al. Serial monitoring of circulating tumor DNA in patients with primary breast cancer for detection of occult metastatic disease. EMBO Mol Med. 2015.

7. Heinz S, Benner C, Spann N, Bertolino E, Lin YC, Laslo P, et al. Simple combinations of lineage-determining transcription factors prime cis-regulatory elements required for macrophage and B cell identities. Mol Cell. 2010;38:576–89.

8 Forbes SA, Beare D, Gunasekaran P, Leung K, Bindal N, Boutselakis H, Ding M, Bamford S, Cole C, Ward S, Kok CY, Jia M, De T, et al. COSMIC: exploring the world's knowledge of somatic mutations in human cancer. Nucleic Acids Res. 2015; 43(Database issue):D805-811

9. Krzywinski M, Schein J, Birol I, Connors J, Gascoyne R, Horsman D, et al. Circos: an information aesthetic for comparative genomics. Genome Research. 2009;19:1639–45.

10. Lemon J. Plotrix: a package in the red light district of R. R-News. 2006;6:8–12.

11. Wolf P, Bielefeld U. aplpack: Another Plot PACKage: stem.leaf, bagplot, faces, spin3R, plotsummary, plothulls, and some slider functions. 2013.
